# Novel Method of Measuring Corneal Viscoelasticity Using the Corvis ST Tonometer

**DOI:** 10.3390/jcm11010261

**Published:** 2022-01-04

**Authors:** Agnieszka Boszczyk, Henryk Kasprzak, Joanna Przeździecka-Dołyk

**Affiliations:** 1 Department of Optics and Photonics, Wroclaw University of Science and Technology, 50-370 Wrocław, Poland; henryk.kasprzak@pwr.edu.pl (H.K.); joanna.przezdziecka-dolyk@pwr.edu.pl (J.P.-D.); 2 Department of Ophthalmology, Wroclaw Medical University, 50-556 Wrocław, Poland

**Keywords:** non-contact tonometry, Corvis ST, corneal applanation, corneal buckling, corneal viscoelasticity

## Abstract

Background: The process of rapid propagation of the corneal deformation in air puff tonometer depends not only on intraocular pressure, but also on the biomechanical properties of the cornea and anterior eye. One of the biomechanical properties of the cornea is viscoelasticity, which is the most visible in its high-speed deformations. It seems reasonable to link the corneal viscoelasticity parameter to two moments of the highest speed of corneal deformations, when the cornea buckles. The aim of this work is to present a method of determining the time and place of occurrence of corneal buckling, examine spatial and temporal dependencies between two corneal applanations and bucklings in the Corvis ST tonometer, and correlate these dependencies with corneal viscoelastic properties. Methods: Images of the horizontal cross section of the Corvis ST deformed cornea from the air puff tonometer Corvis ST were used. 14 volunteers participated in the study, each of them had one eye measured eight times. Mutual changes in the profile slopes of the deformed corneas were numerically determined. They describe pure corneal deformation, eliminating the influence of rotation, and displacement of the entire eyeball. For each point in the central area of the corneal profile, the maximum velocities of mutual slope changes accompanying the applanations were estimated. The times of their occurrence were adopted as buckling times. Results: The propagation of buckling along the corneal profile is presented, as well as the repeatability and mutual correlations between the buckling parameters and intraocular pressure. Based on the relationship between them, a new parameter describing corneal hysteresis: *Corvis Viscoelasticity* (*CVE*) is introduced. It is characterized by high repeatability: *ICC* = 0.82 (0.69–0.93 *CI*) and low and insignificant correlation with intraocular pressure: *r* = 0.25 (*p*-value = 0.38). Conclusion: The results show for the first time how to measure the corneal buckling and viscoelastic effects with Corvis ST. *CVE* is a new proposed biomechanical parameter related to the viscoelastic properties of the cornea, which has high repeatability for the examined subject. The distribution of its values is planned to be tested on different groups of patients in order to investigate its clinical applicability.

## 1. Introduction

Variations in the mutual inclinations of the tangents to the corneal profile are one way to describe corneal deformation. During air puff tonometric measurements, these mutual inclinations are subjected to change, conveying information about pure deformation of the cornea, being insensitive to shifts or rotation of the entire eyeball caused by the air blast. Corneal deformation depends both on the resultant forces that act on it and its biomechanical properties.

The Corvis ST tonometer (OCULUS Optikgeräte GmbH, Wetzlar, Germany) records a sequence of images during each measurement, showing corneal deformations in the horizontal cross-section induced by an air blast. These images can be used to compute the described above mutual slopes of the various corneal regions.

The device provides information on intraocular pressure *IOP*, thickness distribution of the horizontal corneal profile, and a number of dynamic corneal response (*DCR*) parameters that describe the behavior of the cornea during measurement. Taking into account the biomechanical properties of the cornea can be useful as an additional diagnostic tool. For example, the parameters for the dynamic corneal response may indicate the early stage of keratoconus even when tomography and topography appear normal [1]. Furthermore, in normal-tension glaucoma, corneas tend to be softer and more deformable compared to the control group, which is reflected in their dynamic response to air puff [2].

The repeatability of individual parameters and their mutual relationships are important aspects of data analysis. Particular attention is paid to the relationship between the *DCR* parameters and the intraocular pressure. This is because the *IOP* is measured indirectly through the cornea. Its biomechanical properties (thickness, stiffness, and viscoelasticity) can significantly distort the reading. It also works the other way around: due to stresses induced in the viscoelastic cornea by varying intraocular pressure, its biomechanical parameters may change depending on the current *IOP* value [3]. This issue is extremely complex and new models are needed to address it.

The biomechanically corrected intraocular pressure *bIOP* is a characteristic parameter determined by this tonometer. The equation for the *bIOP* value was determined using numerical simulations with the finite element method, taking into account the influence of corneal stiffness, corneal thickness, curvature, and biomechanical properties [4]. It has been shown experimentally that it is in fact less dependent on corneal thickness and age than uncorrected *IOP*, measured with the same tonometer [5,6]. Recently, a material stiffness parameter (Stress-Strain Index *SSI*) has also been proposed, which manifests a high correlation with age and a lack of significant correlation with *IOP* and corneal thickness. Based on the image sequence from the Corvis ST tonometer, *SSI* allows one to estimate the tangent modulus of the cornea under any load or stress [7].

The parameters proposed by the manufacturer are just some of many possible ways of describing, using, or interpreting corneal deflection. However, the complex phenomena that occur during full corneal deflection are not yet fully understood. There is a need to learn more about them, as they can tell more about the properties of the cornea itself, as well as about the entire eye globe or the structures surrounding it.

The corneal stroma consists of about 78% water [8]. Fast deformations of structures containing liquids are accompanied by viscous resistance inside this structure. Viscous resistance depends directly on the velocity of deformation in such a structure [9]. The viscous resistance of cornea can be expected to depend on the velocity of the corneal deformation. During air puff measurements, the fastest deformations inside the cornea appear approximately when the central part of the cornea passes both applanations. This suggests that the corneal viscoelasticity directly affects the way the cornea passes both applanations.

Viscoelastic properties have not yet been described or examined with the Corvis ST tonometer. However, they are determined in the Ocular Response Analyzer (ORA), using *corneal hysteresis CH*, which is linearly dependent on the difference between the air pressures *P*_1_ and *P*_2_ from the jet, during the first and the second applanations [10]. *CH* is used to diagnose and manage suspected glaucoma. It is also useful for monitoring changes, especially in the case of normal tension glaucoma [11]. Some of the recently released articles constate that the viscous properties of the cornea cannot be defined from the air puff applanation. One of these articles suggests that the viscous properties of the cornea did not contribute significantly to the measured displacements [12], but another states that any fast contactless test can provide information on the viscous properties of the cornea [13]. Moreover, Francis et al. directly argue that corneal viscous properties cannot be determined from air puff tonometry [14]. However, this article was commented on by other authors, who stated that hysteresis—an indicator of viscoelasticity—can be determined using both Corvis ST and ORA [15].

Usually, when considering the material viscoelasticity, it is assumed that the material is linearly elastic. A more general approach describes the hyperelastic–viscoelastic material. Such materials are often known as nonlinear viscoelastic materials. It is mentioned in the literature that some biological tissues indicate these hyper-viscoelastic properties. Zhang et al. consider both viscoelasticity and hyperelasticity to establish a more accurate model of the human ear [16]. This approach was also proposed for the material properties of the cornea. Jannesari et al. state that the contribution of material viscosity in the dynamic tonometry test is trivial and can be ignored, but hyper–viscoelastic is a more accurate definition of the behavior of the whole eye globe [17]. The viscoelastic–hyperelastic model of the cornea is examined in papers [18,19]. However, examinations of both models were carried out on ex vivo porcine and human corneas, during relatively slow, monotonic loading, and deformations. Corneal deformations during real-time air puff tonometry are significantly faster and distinctly nonmonotonic.

The process of very fast corneal applanation is very complex and is not well recognized from a mechanical point of view. Theoretical and experimental analysis of the complex structure, quasi-spherical, viscoelastic shell buckling would be essential and very interesting for modeling and investigating corneal buckling during its air puff applanation. Some papers concern buckling of the elastic spherical shell [20]. However, to the best of our knowledge, there are no such papers concerning the viscoelastic shell.

In the paper [21], both corneal applanations in ORA are examined and the corneal buckling process is described and discussed. It draws attention that the times referring to both bucklings *t*_A_ and *t*_B_ are very close to both applanation times *t*_A1_ and *t*_A2_, but they are not precisely the same.

Numerical analysis of the dynamics of the corneal profile inclinations observed in image sequences from Corvis ST may enable the identification of two-time moments, when the two mutual inclinations of the corneal profile in the area of the apex are extremely fast. If these time moments are very close to times of the first and the second applanations, they can be assumed to be the times of both corneal bucklings. Since buckling of the central cornea due to air blast is associated with a sudden and rapid redistribution of stresses inside the cornea, it is likely that it can also be observed in Corvis ST measurements during both applanations.

The aim of this work is to present the method for determining the time and place of occurrence of corneal buckling, to examine spatial and temporal dependencies between the two corneal applanations and the bucklings in the Corvis ST tonometer, and to correlate these dependencies with corneal viscoelastic properties. The proposed parameters may become an alternative to corneal hysteresis measured by ORA, achievable in the measurements with Corvis ST.

## 2. Materials and Methods

These retrospective data were collected during a previous study, conducted at an outpatient unit of the Department of Ophthalmology of the Medical University Hospital in Wroclaw, Poland, with the prior consent of the bioethics committee. We did not perform the sample size calculation, as this is the pilot study that we intend to use in the future. Based on this perspective, it was deemed important to assess the possible best-choice parameters on the smaller group, as the analytical approach is a time- and resource-consuming procedure that requires several iterations of the process. Fourteen healthy volunteers participated in this study (the average age of the participants was 28 years with a standard deviation of ±11). The tested group included healthy Caucasian men and women, without diagnosed eye diseases, symptoms, or previous eye surgery. They were examined using the noncontact Corvis ST tonometer (OCULUS Optikgeräte GmbH). During each measurement, the tonometer was set in the appropriate position just in front of the subject’s eye, according to the instructions displayed on the device screen. A blast of air was released from the device nozzle to deflect the cornea.

Each person had performed eight measurements on one selected eye, on the same day, one after the other, several minutes apart. Subjects were encouraged to relax and move their heads between subsequent measurements. The whole procedure for a single eye lasted about 30 min. These multiple measurements were taken on seven right eyes and seven left eyes. The allocation of people to the left or right eye measurement group was random (ocular dominance was not taken into account). All subjects were informed about related procedures and consented to the examination. The study was carried out according to the Declaration of Helsinki and obtained ethical approval.

During each measurement, the device allowed the registration of a sequence of 140 images (200 × 576 pixels) displaying a horizontal cross-section of a deforming cornea. Each sequence lasted about 32 ms. The size of a pixel was assumed to be 0.016 × 0.016 mm [22]. A total of 112 image sequences were exported using the Corvis ST software (version 1.3r1727) and processed using Matlab.

First, horizontal profiles of the examined corneas were detected, as described in detail in [23]. As a result, for each measurement, two-dimensional matrices M(i,k) were obtained (number of images i from 1 to 140, which corresponds to the period from 0 to 32 ms; image columns *k* from 1 to 576). These matrices were smoothed using a 5 × 7 window size averaging filter to eliminate possible disturbances. This smoothing procedure created a matrix of smoothed data Ms(i,k), describing the height of the corneal profile in image number i and image column *k*.

Local slopes α of the corneal profiles were calculated as follows:

(1) $$\alpha \left(i,k\right)=\mathrm{atan}\left(\frac{dMs\left(i,k\right)}{dk}\right)$$

where $\frac{d}{dk}$ is a five-point numerical derivative of the coordinate (column) *k*.

Slope changes, which means slope variation relative to the initial state in the first frame, were calculated according to the following formula:

(2) Δα(i,k)=α(i,k)−α(1,k).

To remove the influence of possible eye rotation on the analyzed values of the slope along the corneal profile, the change in the mutual slope at two points located symmetrically to the apex: *Profile Slope Variations* (*PSV*), were determined as follows

(3) PSV(i)=Δα(i, k)−Δα(i, 576−k).

**Figure 1:**
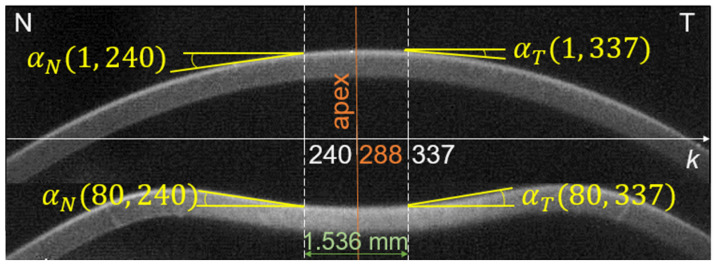
Local slope of the corneal profile α in the image columns *k* = 240 and 337 (points symmetrical to the apex on the nasal *N* and temporal *T* sides) at the beginning of the measurement (top image *i* = 1) and during the deformation (bottom image *i* = 80).

Since the largest changes due to air blast are observed in the central part of the cornea, our further analysis focused on this area. Two points located approximately 0.768 mm from the center on the nasal and temporal side were selected (corneal profile points falling on the 240th and 337th columns of images—see Figure 1). This choice has been made due to the high reproducibility and correlations of the proposed parameters referred to these points. Such a distance from the apex is large enough for clear observation of the mutual slope changes (low risk of numerical errors caused by too small mutual slope changes), yet much less than the minimum applanation lengths or peak distance along the corneal profile indicated by the tonometer software.

**Figure 2:**
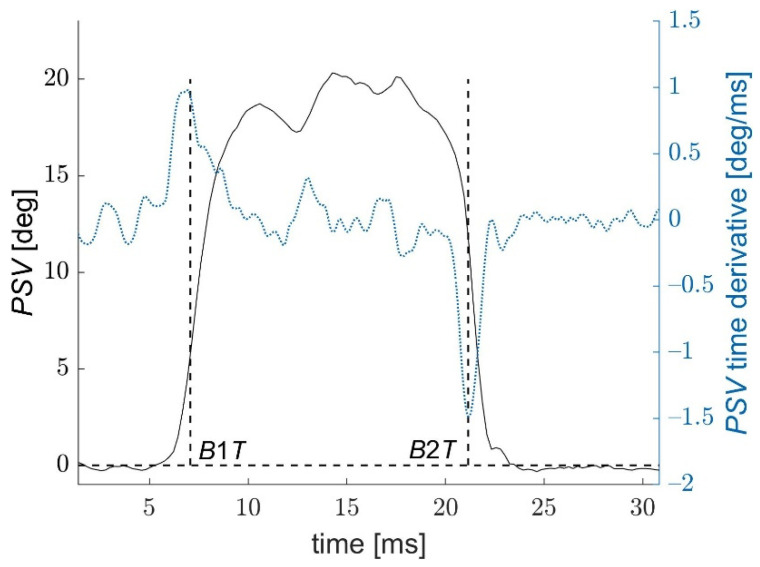
Local *Profile Slope Variations* for a corneal profile point per 240th column of the image sequences. *B*1*T* and *B*2*T* describe two times, when *PSV* achieve the fastest variations (values of the first time-derivative of *PSV* are extreme).

Figure 2 shows an example of a *PSV* function during measurement. In the time moments close to the applanation, *PSV* dynamically changes its value, while in the remaining moments of the measurement, it fluctuates around certain values. 

After a tenfold densification of the *PSV* function points, with the smoothing spline method, the *PSV* time derivative was calculated. Two moments *B*1*T* and *B*2*T* close to the applanation times, in which the velocity (derivative) of the *PSV* reaches two extreme values *DSV*1 and *DSV*2 were determined. Since times *B*1*T* and *B*2*T* are characterized by the fastest possible corneal deformations (the fastest changes in the local corneal slope), one can assume that they correspond to the times when the complex corneal structure loses its stability under extremely fast loading and starts its buckling.

**Figure 3:**
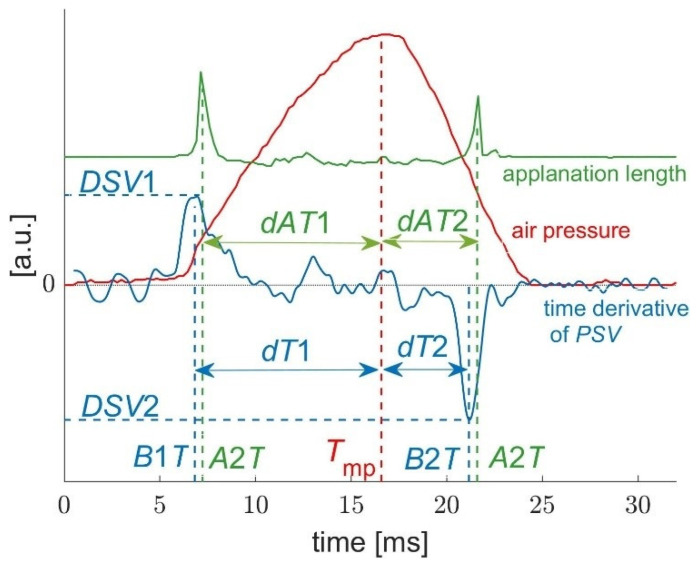
Three curves from an exemplary measurement (nozzle air pressure, applanation length, and the time derivative of the *PSV* function from Figure 2) and related parameters.

The times of applanations A and buckling B are very close to each other, but they do not coincide (as shown in Figure 3). The corresponding differences in both times are marked as *D*1*T* and *D*2*T*:

(4) D1T=A1T−B1T,

(5) D2T=A2T−B2T.

Additionally, the parameters analyzed for each measurement were (Figure 3):

- *T*_mp_—time of the maximum air pressure from the nozzle. This parameter was determined on the basis of air pressure diagrams exported from the tonometer software. As the air pressure curves were very similar to each other, *T*_mp_ differed only slightly between measurements and was in the range of 16.23–16.72 ms;
- *dT*1 and *dT*2—time periods from *B*1*T* to *T*_mp_ and from *T*_mp_ to *B*2*T*;
- *dAT*1 and *dAT*2—time differences *T*_mp_—*A*1*T* and *A*2*T*—*T*_mp_.

Some algebraic operations between buckling parameters represent the asymmetry in the dynamical response of the cornea during its loading and unloading, which is related to the hysteresis of the corneal response. This paper presents the results of the analysis for the following parameters: $\left|DSV1\right|+\left|DSV2\right|,\;D1T-D2T,\;D1T+D2T,\;\;\frac{dT1}{dT2}$, $\frac{dAT1}{dAT2}$, $\frac{D1T}{dT1}$, $\frac{D2T}{dT2}$, $\frac{D1T}{dT1}+\frac{D2T}{dT2}$.

A further step was the correlation analysis between the parameters introduced above and with the parameters obtained from the Corvis ST software, such as *IOP*—noncorrected intraocular pressure, *bIOP*—biomechanically corrected intraocular pressure, *CCT*—central corneal thickness, *A*1*T* and *A*2*T*—times of the first and second applanation, *A*1*L* and *A*2*L*—lengths of the first and the second applanation.

### Statistical Analysis

The ranges and repeatability of the basic parameters obtained from Corvis ST and the corneal slope parameters were examined. For this purpose, the following quantifiers were determined for all eyes measured: mean values, standard deviations, and intraclass correlation coefficient *ICC* [24] coefficient of repeatability *CR* [25], coefficient of variation *CV*. In addition, the incidence of significant relationships between selected parameters was also estimated. Based on the mean values for individual eyes, the ’s correlation coefficients *r* for pairs of different variables were calculated [26]. The adopted significance level was 0.05.

## 3. Results

**Figure 4:**
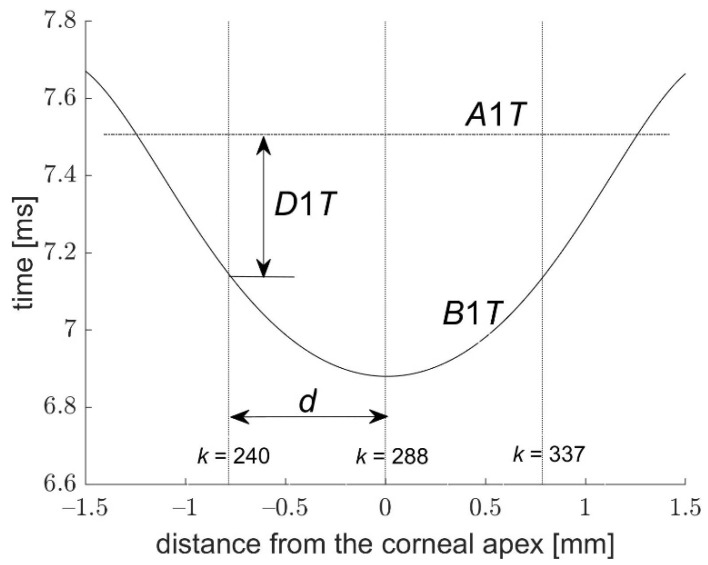
Times *B*1*T* and *D*1*T* as a function of the distance from the corneal center; *d*—the distance where the slope variations were analyzed.

**Figure 5:**
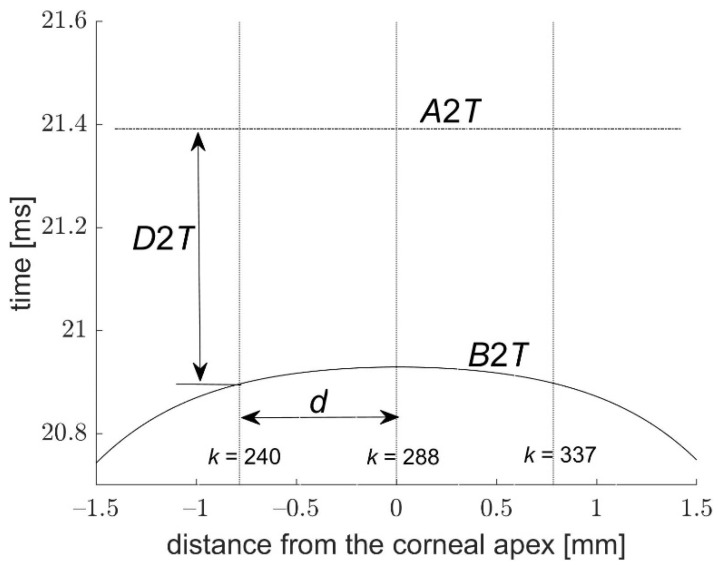
Times *B*2*T* and *D*2*T* as a function of the distance from the corneal center; *d*—the distance where the slope variations were analyzed.

Figure 4 and Figure 5 present the graphs of dependencies between *D*1*T* and *B*1*T*, as well as between *D*2*T* and *B*2*T* from one measurement as a function of the distance *d* from the corneal center. The courses of the first buckling *B*1*T* and the second *B*2*T* in both figures show that they appear at different times depending on the distance from the corneal center. Analysis of these dependencies was carried out before more extensive calculations. In case of the distance closer to the corneal center, the slope variations and their angular velocity *DSV*1 and *DSV*2 are ignorable. On the other hand, when the distance *d* is too high, the analyzed area is no longer related to the deflected corneal center. Therefore, the time difference *D*1*T* becomes very small and then negative. In this situation, obtained results characterize a low repeatability of the analyzed parameters. It should be noted that it is possible to find a distance *d* that is large enough for *B*1*T* to occur later than *A*1*T* (*d* comparable to the length of the first application or greater). Furthermore, *B*2*T* always occurs before *A*2*T*, regardless of the distance of the analyzed point of the cornea from the apex.

It is interesting to note that the first buckling starts before the first applanation in the corneal center and expands in time radially from the center. In the case of the second buckling (Figure 5), it falls in the center of the cornea also before the second applanation. The dispersal of the first buckling from the corneal center before the first applanation is slower than the descent of the second buckling to the center of the cornea before the second applanation, while the average time delay between the first buckling and the first applanation *D*1*T* is smaller than the similar delay *D*2*T* during the second applanation.

The corneal applanations and the fastest deformations of the corneal profile in the distance applied in the paper (48 px from the apex) appear clearly at different times for both applanations. Interestingly, in each of the analyzed measurements, buckling always occurred right before the corresponding applanation. Detailed descriptive statistics of the parameters considered can be seen in Table 1.

Analysis of correlations between considered parameters showed interesting and important properties, which enables a new approach to Corvis ST measurements. We decided to split the analyzed correlation coefficients into two characteristic groups of parameters.

The first group contains parameters highly correlated with *IOP*. They are mainly defined by both applanation times, buckling times, but also by difference *D*1*T − D*2*T* as well as by both extreme speeds of the slope variations *DSV*1 and *DSV*2. The mutual correlations between them can be seen in Table 2.

**Figure 6:**
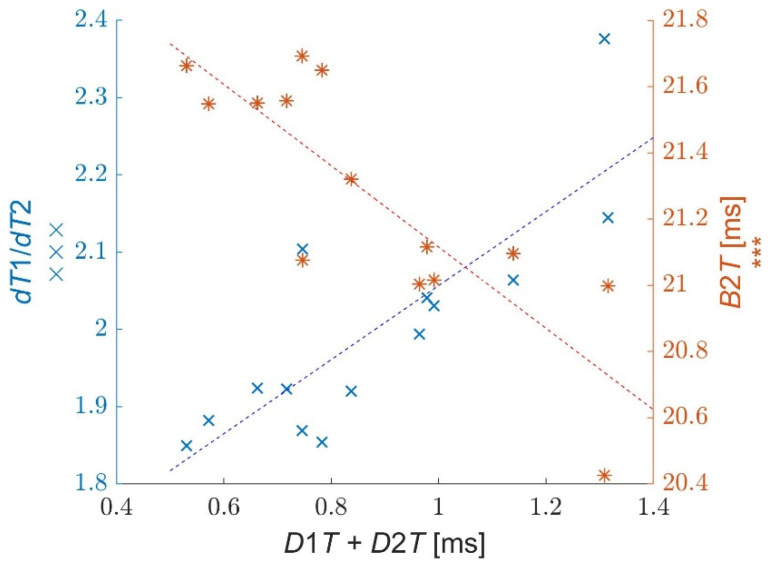
Correlations of parameters $\frac{dT1}{dT2}$ (×) and *B*2*T* (*) vs. sum *D*2*T* + *D*1*T*.

The second group consists of the time *B*2*T* and the relations between *D*1*T*, *D*2*T*, *dT*1*,* and *dT*2. The main property of these parameters is their high mutual correlation related to the viscoelastic properties of the cornea and low correlations with *IOP*. The mutual correlations between these parameters can be seen in Table 3. An exemplary correlation plot is presented in Figure 6.

Due to the small values of *D*1*T* and *D*2*T*, the values of some parameter pairs presented in Table 2 and Table 3 are very similar to each other, such as *A*1*T* and *B*1*T*, *A*2*T* and *B*2*T*, *dT*1/*dT*2 and *dAT*1/*dAT*2. However, one can see that the time parameters related to both applanations are higher correlated with *IOP*, while the time parameters referred to both bucklings have better correlations with viscoelastic properties. A good example is the correlation coefficient between *A*1*T* and *IOP* (*r* = 0.99), while a similar correlation between *B*1*T* and *IOP* is smaller (*r* = 0.79). On the other hand, the correlation between *dT*1/*dT*2 and *B*2*T* is higher (*r* = −0.94) than the correlation between *dAT*1/*dAT*2 and *A*2*T* (*r* = −0.78). There are more such observations among the respective data, which show that it is particularly important to take into account the appropriate parameters among similar ones during the calculation of *IOP* and *bIOP* or the calculation of parameters referring to viscoelastic properties.

**Figure 7:**
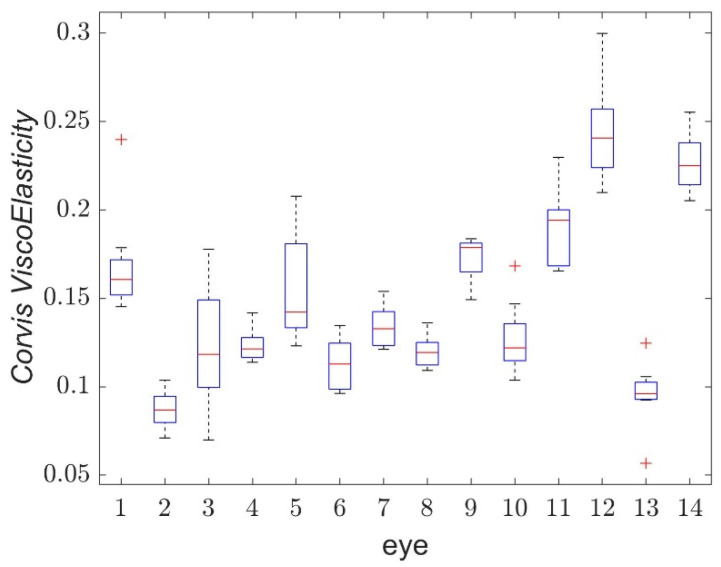
Boxplot of *Corvis ViscoElasticity* for single eyes of measured volunteers.

Analysis of the mutual correlations presented in Table 3 enables the proposal of a new parameter, which characterizes the viscoelastic properties of the cornea. We propose to use the parameter $\frac{D1T}{dT1}+\frac{D2T}{dT2}$, describing asymmetry of corneal behavior during its loading and unloading, using the characteristic relation between buckling and applanation moments. The selected parameter has high repeatability (*ICC* = 0.82) and its correlations with other parameters from the table are also high. This parameter is also dimensionless, which can be its advantage. To avoid its complex notation, we propose to call this parameter *CVE* (*Corvis ViscoElasticity*). The ranges of the *CVE* values for individual eyes are shown in Figure 7.

To analyze the property of two applanation lengths *A*1*L* and *A*2*L* in the Corvis ST tonometer, their repeatabilities and correlations with other parameters considered were calculated and examined. Repeatability coefficients for both applanation lengths were very low. Several of the parameters analyzed showed a correlation coefficient with *A*1*L* greater than 0.7 (Table 4). The highest correlation appears for the ratio $\frac{D1T}{D2T}$ and amounts 0.85. There was a problem to find a high correlation of the second applanation length *A*2*L* with any of the parameters considered.

## 4. Discussion

The results presented in this paper enable the description and analysis of corneal buckling in measurements using the Corvis ST tonometer. The main areas of interest were processes related to the first and the second applanations, the values and times of extreme angular speeds of the corneal slope variations, and the asymmetry of both applanations in relation to the maximum air pressure from the jet. Extreme speeds of corneal slope variations appear directly before the first and the second applanations, not during these applanations, as expected. Such extreme speeds of variation of the local corneal slope indicate its local buckling. As was presented in both the paper [21] and in this study, buckling effects did not appear directly during both corneal applanations but very close to these flattenings of the cornea. Thus, it is interesting and important to elucidate the reason for this effect and the dependence of the time difference between these processes and the corneal properties.

From a hydrodynamic point of view, a rapid deformation of structures consisting mainly of liquid causes a viscosity effect, which is proportional to the velocity of the deformation or to its power (nonlinear effect). Since the cornea consists mainly of water, one has to expect the prevalence of viscoelastic effect (viscoelastic resistance) during fast corneal deformations, which appear mainly during the time periods of both applanations. The magnitude of this effect depends on the velocity of deformation of the structure but also on the properties of the structure, described by some characteristic constants. This approach suggests correlating the viscoelastic properties of the cornea (characterized by a constant) with the time delay between the corneal applanation and corneal buckling. The magnitude of this delay should be a function of the corneal structure resistance caused by its viscoelasticity. Numerical analysis of the results obtained showed that there are quite high correlations between the selected parameters related to the mentioned time delays (*D*1*T* and *D*2*T*) and very low correlations with *IOP*.

An interesting correlation appears between both buckling times *B*1*T* and *B*2*T* vs. *D*1*T*. No correlation between *D*1*T* and *B*1*T* means that the time of the first buckling *B*1*T* (and the time of the first applanation *A*1*T*) does not have any influence on their difference *D*1*T*. Both *A*1*T* and *B*1*T* times depend mainly on the *IOP* behind the cornea. However, unexpectedly high correlations were obtained between the time difference *D*1*T* during the first applanation and the second buckling time *B*2*T* as well as the second applanation *A*2*T*. This indicates that processes between the first buckling and the first applanation determine the times when the second buckling and the second applanation appear. This effect can be explained by the fact that the two intervals *D*1*T* and *D*2*T* characterize the viscoelastic properties of the cornea, due to rapid processes within these intervals. Such properties also determine time intervals between applanations (*r* = −0.71 for *D*1*T* vs. *A*2*T* − *A*1*T*) and bucklings (*r* = −0.70 for *D*1*T* vs. *B*2*T* − *B*1*T*). The sum of times *D*1*T* and *D*2*T* underlines the viscoelastic properties of the cornea, while their difference reduces these properties being more related to *IOP*. Here, some far-reaching analogies can be seen with Ocular Response Analyzer (ORA) measurements, where the sum of air pressures during corneal applanations *P*1 and *P*2 is proportional to the *IOP* value, and the difference of both pressures is proportional to *Corneal Hysteresis CH* [27,28].

Furthermore, it appears that the parameters that describe the asymmetry of both bucklings $\frac{dT1}{dT2}$ and applanations $\frac{dAT1}{dAT2}$ in relation to the time of maximum air pressure *T*_mp_ are somehow correlated with the parameters associated to the corneal viscoelastic properties. A similar effect appears in the ORA device. According to our earlier examinations, the correlation coefficient of corneal hysteresis *CH* with the similar asymmetry $\frac{dAT1}{dAT2}\;$ of both applanations falls within the range 0.75–0.85. However, in ORA measurements, *dAT*1 is smaller than *dAT*2, opposite to Corvis ST measurements.

Some authors affirm that the viscous properties of the cornea do not contribute significantly to the measured displacements in air puff tonometers or even that they cannot be determined [12,14]. They refer to in vitro measurements on a slowly and monotonically deformed cornea. The average viscous properties of the cornea under such slow deformations probably demonstrate a very small influence on the results obtained. However, in the case of rapid variability in deformation during an extremely short period, when buckling and applanation of the cornea appear within a fraction of milliseconds, the viscous effect can be clearly observed and measured. This effect is most likely nonlinear and is clearly observed only during a quick variation of corneal deformation.

The applanation of the cornea means the flattening of the first corneal surface. Taking into account the increase in corneal thickness outside its center, the flat anterior surface of the cornea means that the borders of the deeper-located layers of the corneal structure are still convex. Due to the very complex corneal structure, it is difficult to describe how the mentioned borders pass their flattening, and how an extremely fast buckling propagates inside a complex, multilayered, anisotropic structure. Moreover, corneal applanation cannot indicate an ideally flat area of the central applanation. There are always rapid vibrations or small irregularities on this surface. Therefore, according to the article [21] the question of ‘quality of both corneal applanations’ is still open. It cannot be excluded that various corneas are characterized by various ‘qualities of applanation’ or areas of applanation. The presented correlation coefficients with the first applanation length *A*1*L* suggest that this parameter can be somehow related to other examined parameters.

One of the limitations of the study is that it was performed on a small sample of healthy eyes, so it does not provide much information about expected values of population parameters. We are aware that the study sample size should be larger to increase the validity of the new proposed method. Now, with the direction of further research already established, we plan to test the method in larger groups of subjects, both healthy and suffering from some specific pathologies. On the other hand, the average values of the parameters read from the tonometer software and their repeatability are comparable to the values presented in studies using larger study groups [29,30].

Another limitation was that the Corvis ST tonometer cannot change the characteristics of the air pulse—its length and pressure. These parameters are constant and repeatable in subsequent measurements, and therefore it is not possible to examine their influence on the considered parameters. In addition, it would be good to model the buckling in the cornea and adjust the mechanical parameters of the model to reflect its actual behavior. However, until an exact model is not available, this step cannot be accomplished.

The other possible drawback of the study is the fact that we based only on the commercially available devices. However, scientific research is carried out on OCT devices to record the reaction of eye structures to a blast of air [31]. Possibly, the parameters related to corneal buckling could also be determined with this newly developed device.

Therefore, in order to unify the measurement results obtained on each of these devices, their different air impulse characteristics as well as different methods of recording the dynamic reaction of the cornea (corneal reflection light detector, Scheimpflug images or OCT) should be taken into account.

During the numerical analysis of the repeatability of the parameters and their mutual correlations, the influence of the *Central Corneal Thickness CCT* on the values obtained was also examined. The parameters were multiplied or divided by *CCT* and then analyzed. In some cases, such an operation increases the parameter repeatability, and in other cases increases their mutual correlation. However, this influence of *CCT* on the results received was not very clear and unique. More detailed analysis of this effect could be carried out in the next examinations.

The use of Corvis ST to measure dynamic changes of the cornea allows for a non-invasive study of the biomechanical properties of the eye. We believe that the new parameters introduced, which show a high repeatability and very low correlation with intraocular pressure, are highly correlated with the viscoelastic properties of the cornea during applanation. The proposed *CVE* parameter may find application in the diagnosis of the early stage of corneal keratoconus, the variation of corneal properties due to glaucoma, the effect of corneal crosslinking, or the influence of corneal viscoelastic properties on different refractive surgery procedures.

*CVE* describes the viscoelastic properties of the cornea, in a way similar to the *CH* hysteresis in ORA. However, this is not exactly the same case because even the *CVE* and *CH* units of measurement are different. Moreover, the variable, nonlinear viscoelasticity of the medium (as is the case with the cornea) can be described by different parameters, so that *CVE* and *CH* are not exactly the same. It may be that *CVE* is better at distinguishing one specific eye pathology and *CH* is better at distinguishing other pathologies. In the next step, it would be interesting to compare the *CVE* and *CH* of the same corneas, in individuals who are both healthy and with pathologies (especially glaucoma).

After examination of the characteristic values for given groups of patients, the proposed parameters can become an additional tool for ophthalmic diagnosis, which will be simple, fast, and non-invasive. In addition, the presented method of buckling determination may be useful in the creation and validation of a mechanical model of the cornea.

## 5. Conclusions

According to the best knowledge of the authors, the presented results of in vivo experiments on the eyes of healthy volunteers show for the first time that the corneal buckling effect can be observed and measured on the Corvis ST tonometer. Fine numerical analysis of the corneal profile obtained from each of the 140 video frames and examination of the dynamics of local slope variations of the profile enables a quantitative description of both buckling time moments. Some new parameters were defined and proposed regarding the processes of both bucklings and applanations. In particular, a specific parameter *CVE* was proposed to determine the viscous properties of the cornea due to its high reproducibility (*ICC* = 0.82), dimensionlessness, and high correlations with other parameters related to corneal viscoelasticity. Differences between the magnitude of this parameter for different corneas can be a useful indicator of the characteristic biomechanical feature of an individual cornea. The paper may be a prelude to the application of the proposed analysis to pathological corneas and, more generally, to an investigation of the corneal viscous properties.

## Figures and Tables

**Table 1:** The mean values of the considered parameters, their standard deviations (SD), ranges, as well as measures of their repeatability in the form of coefficient of repeatability (CR), coefficient of variation (CV), and intraclass correlation coefficient (ICC) with 95% confidence interval (CI); u—the unit of a given parameter.

Parameters		*ICC* (95% *CI*)	CR [u]	CV [%]	Mean [u]	SD [u]	Range [u]
Corvis ST software	IOP [mmHg]	0.77 (0.62–0.9)	3.2	7.5	15.5	2.2	10–21
	bIOP [mmHg]	0.73 (0.56–0.88)	2.9	7.1	14.8	1.8	10.3–19.7
	A1T [ms]	0.79 (0.64–0.91)	0.32	1.5	7.38	0.23	6.86–7.92
	A2T [ms]	0.69 (0.51–0.86)	0.52	0.85	21.8	0.3	21.1–22.5
	A1L [mm]	0.12 (0–0.37)	0.6	8.4	2.56	0.11	1.52–3.13
	A2L [mm]	0.068 (0–0.28)	3.1	31	3.6	0.5	0.5–6.6
	CCT [μm]	0.97 (0.94–0.99)	18	1.2	562	38	492–630
New proposed dynamical parameters	B1T [ms]	0.76 (0.6–0.9)	0.28	1.5	7.02	0.19	6.47–7.46
	B2T [ms]	0.79 (0.64–0.91)	0.51	0.87	21.3	0.36	20.2–22
	D1T [ms]	0.78 (0.62–0.91)	0.20	20	0.36	0.14	0.10–0.72
	D2T [ms]	0.80 (0.65–0.92)	0.18	13	0.52	0.13	0.24–0.90
	dT1 [ms]	0.59 (0.39–0.8)	0.39	1.5	9.47	0.18	8.93–9.92
	dT2 [ms]	0.74 (0.57–0.89)	0.54	4.1	4.77	0.34	3.75–5.50
	dAT1 [ms]	0.74 (0.57–0.88)	0.4	1.6	9.11	0.25	8.49–9.70
	dAT2 [ms]	0.65 (0.46–0.84)	0.53	3.6	5.29	0.27	4.54–6.05
	DSV1 [deg/ms]	0.85 (0.74–0.94)	0.43	6.4	2.40	0.38	1.51–3.15
	DSV2 [deg/ms]	0.82 (0.69–0.93)	0.39	4.5	−3.12	0.30	−3.87–−2.14
	$\left|DSV1\left|+\right|DSV2\right|\;\left[\frac{\deg}{\mathrm{ms}}\right]$	0.89 (0.8–0.96)	0.63	4.1	5.52	0.66	3.78–6.86
	D1T+D2T [ms]	0.8 (0.65–0.92)	0.34	14	0.88	0.25	0.36–1.53
	D1T−D2T [ms]	0.73 (0.56–0.88)	0.18	42	−0.16	0.11	−0.35–0.20
	dT1/dT2	0.75 (0.59–0.89)	0.22	4	2.00	0.14	1.73–2.5
	dAT1/dAT2	0.57 (0.37–0.79)	0.15	3.1	1.73	0.06	1.52–1.92
	D1T/dT1	0.79 (0.64–0.91)	0.021	20	0.038	0.015	0.011–0.077
	D2T/dT2	0.83 (0.69–0.93)	0.044	14	0.111	0.035	0.044–0.228
	CVE	0.82 (0.69–0.93)	0.059	14	0.149	0.047	0.057–0.300

**Table 2:** Pearson’s correlation coefficients *r* with *p*-values (in parentheses) between selected parameters related mainly to both applanation times, *D*1*T* and *D*2*T* times, and extremal profile slope velocities, *DSV*1 and *DSV*2. The parameters in the table were selected to have high correlation with *IOP*.

*r* (*p*-Value)	IOP	bIOP	A1T	B1T	A2T	B2T	D1T−D2T	DSV1
dAT1	−0.95 (<0.001)	−0.88 (<0.001)	−0.96 (<0.001)	−0.62 (0.018)	0.81 (<0.001)	0.67 (0.009)	−0.8 (<0.001)	0.89 (<0.001)
dAT2	−0.79 (<0.001)	−0.81 (<0.001)	−0.78 (<0.001)	−0.49 (0.078)	0.97 (<0.001)	0.88 (<0.001)	−0.41 (0.15)	0.71 (0.005)
A1T	0.99 (<0.001)	0.92 (<0.001)		0.79 (<0.001)	−0.76 (0.002)	−0.57 (0.032)	0.8 (<0.001)	−0.93 (<0.001)
A2T	−0.76 (0.002)	−0.79 (<0.001)	−0.76 (0.002)	−0.37 (0.2)		0.94 (<0.001)	−0.43 (0.12)	0.69 (0.007)
A2T−A1T	−0.92 (<0.001)	−0.9 (<0.001)	−0.92 (<0.001)	−0.59 (0.027)	0.95 (<0.001)	0.83 (<0.001)	−0.63 (0.015)	0.84 (<0.001)
D1T−D2T	0.8 (<0.001)	0.65 (0.012)	0.8 (<0.001)	0.63 (0.016)	−0.43 (0.12)	−0.21 (0.47)		−0.9 (<0.001)
DSV1	−0.95 (<0.001)	−0.8 (<0.001)	−0.93 (<0.001)	−0.76 (0.002)	0.69 (0.007)	0.46 (0.1)	−0.9 (<0.001)	
DSV2	0.75 (0.002)	0.56 (0.037)	0.73 (0.003)	0.54 (0.049)	−0.43 (0.13)	−0.23 (0.43)	0.94 (<0.001)	−0.86 (<0.001)
|DSV1|+|DSV2|	−0.89 (<0.001)	−0.72 (0.004)	−0.87 (<0.001)	−0.68 (0.007)	0.59 (0.026)	0.37 (0.2)	−0.95 (<0.001)	0.97 (<0.001)

**Table 3:** Pearson’s correlation coefficients *r* with *p*-values (in parentheses) between selected parameters related to both buckling times and extremal profile slope velocities. Presented parameters have low or no correlation with *IOP*. They characterize the viscoelastic properties of the cornea. Bold numbers determine correlation coefficients of parameters vs. selected parameter *CVE* ($\frac{D1T}{dT1}+\frac{D2T}{dT2}$), having the highest reproducibility coefficient (*ICC*).

*r* (*p*-Value)	$\frac{D1T}{dT1}$	$\frac{D2T}{dT2}$	$\frac{dT1}{dT2}$	CVE	*B2T*	IOP
D1T+D2T	0.9 (<0.001)	0.94 (<0.001)	0.82 (<0.001)	**0.99** **(<0.001)**	−0.84 (<0.001)	0.26 (0.38)
$\frac{D1T}{dT1}$		0.72 (0.0037)	0.73 (0.003)	**0.85** **(<0.001)**	−0.84 (<0.001)	0.59 (0.028)
$\frac{D2T}{dT2}$			0.89 (<0.001)	**0.98** **(<0.001)**	−0.82 (<0.001)	0.09 (0.76)
$\frac{dT1}{dT2}$				**0.89** **(<0.001)**	−0.95 (<0.001)	0.4 (0.16)
CVE					**−0.88** **(<0.001)**	**0.25** **(0.38)**

**Table 4:** Pearson’s correlation coefficients *r* higher than 0.7 between the first applanation length *A*1*L* and the selected parameters. The correlation *p*-values are placed in parentheses.

*r* (*p*-Value)	*IOP*	*A*1*T*	*D*1*T*	$\frac{D1T}{D2T}$	*D*1*T − D*2*T*	*DSV*1
*A*1*L*	0.72 (0.004)	0.72 (0.004)	0.76 (0.002)	0.85 (<0.001)	0.78 (0.001)	−0.74 (0.003)

## Data Availability

The data presented in this study are available on request from the corresponding author.

## References

[1] Vinciguerra R., Ambrósio R., Roberts C.J., Azzolini C., Vinciguerra P. (2017). Biomechanical Characterization of Subclinical Keratoconus Without Topographic or Tomographic Abnormalities. J. Refract. Surg..

[2] Vinciguerra R., Rehman S., Vallabh N.A., Batterbury M., Czanner G., Choudhary A., Cheeseman R., Elsheikh A., Willoughby C.E. (2020). Corneal Biomechanics and Biomechanically Corrected Intraocular Pressure in Primary Open-Angle Glaucoma, Ocular Hypertension and Controls. Br. J. Ophthalmol..

[3] Vinciguerra R., Elsheikh A., Roberts C.J., Ambrósio R., Kang D.S.Y., Lopes B.T., Morenghi E., Azzolini C., Vinciguerra P. (2016). Influence of Pachymetry and Intraocular Pressure on Dynamic Corneal Response Parameters in Healthy Patients. J. Refract. Surg..

[4] Elsheikh A., Mohammadvali A., Chen K.-J. (2016). Biomechanically Corrected IOP Measurement. Highlights Ophthalmol..

[5] Bao F.J., Huang Z.X., Huang J.H., Wang J.J., Deng M.L., Li L.N., Yu A.Y., Wang Q.M., Elsheikh A. (2016). Clinical Evaluation of Methods to Correct Intraocular Pressure Measurements by the Goldmann Applanation Tonometer, Ocular Response Analyzer, and Corvis ST Tonometer for the Effects of Corneal Stiffness Parameters. J. Glaucoma.

[6] Eliasy A., Chen K.-J., Vinciguerra R., Maklad O., Vinciguerra P., Ambrósio R., Roberts C.J., Elsheikh A. (2018). Ex-Vivo Experimental Validation of Biomechanically-Corrected Intraocular Pressure Measurements on Human Eyes Using the CorVis ST. Exp. Eye Res..

[7] Eliasy A., Chen K.-J., Vinciguerra R., Lopes B.T., Abass A., Vinciguerra P., Ambrósio R., Roberts C.J., Elsheikh A. (2019). Determination of Corneal Biomechanical Behavior In-Vivo for Healthy Eyes Using CorVis ST Tonometry: Stress-Strain Index. Front. Bioeng. Biotechnol..

[8] Dupps W.J., Wilson S.E. (2006). Biomechanics and Wound Healing in the Cornea. Exp. Eye Res..

[9] Falkovich G. (2018). Fluid Mechanics.

[10] Terai N., Raiskup F., Haustein M., Pillunat L.E., Spoerl E. (2012). Identification of Biomechanical Properties of the Cornea: The Ocular Response Analyzer. Curr. Eye Res..

[11] Morita T., Shoji N., Kamiya K., Fujimura F., Shimizu K. (2012). Corneal Biomechanical Properties in Normal-Tension Glaucoma. Acta Ophthalmol..

[12] Roy S.A., Kurian M., Matalia H., Shetty R. (2015). Air-Puff Associated Quantification of Non-Linear Biomechanical Properties of the Human Cornea in Vivo. J. Mech. Behav. Biomed. Mater..

[13] Simonini I., Angelillo M., Pandolfi A. (2016). Theoretical and Numerical Analysis of the Corneal Air Puff Test. J. Mech. Phys. Solids.

[14] Francis M., Matalia H., Nuijts R.M.M.A., Haex B., Shetty R., Roy S.A. (2019). Corneal Viscous Properties Cannot Be Determined from Air-Puff Applanation. J. Refract. Surg..

[15] Abass A., Roberts C.J., Lopes B., Eliasy A., Vinciguerra R., Ambrósio R., Vinciguerra P., Elsheikh A. (2020). Can the Corvis ST Estimate Corneal Viscoelasticity?. J. Refract. Surg..

[16] Zhang J., Jiao C., Zou D., Ta N., Rao Z. (2020). Assigning Viscoelastic and Hyperelastic Properties to the Middle-Ear Soft Tissues for Sound Transmission. Biomech. Model. Mechanobiol..

[17] Jannesari M., Mosaddegh P., Kadkhodaei M., Kasprzak H., Jabbarvand Behrouz M. (2019). Numerical and Clinical Investigation on the Material Model of the Cornea in Corvis Tonometry Tests: Differentiation between Hyperelasticity and Viscoelasticity. Mech. Time-Depend. Mater..

[18] Su P., Yang Y., Xiao J., Song Y. (2015). Corneal Hyper-Viscoelastic Model: Derivations, Experiments, and Simulations. Acta Bioeng. Biomech..

[19] Whitford C., Movchan N.V., Studer H., Elsheikh A. (2018). A Viscoelastic Anisotropic Hyperelastic Constitutive Model of the Human Cornea. Biomech. Model. Mechanobiol..

[20] Audoly B., Hutchinson J.W. (2020). Localization in Spherical Shell Buckling. J. Mech. Phys. Solids.

[21] Jóźwik A., Kasprzak H., Kozakiewicz A. (2019). Corneal Buckling during Applanation and Its Effect on the Air Pressure Curve in Ocular Response Analyzer. Int. J. Environ. Res. Public Health.

[22] (2016). User Guide Corvis^®^ ST (G/72100/0117/en), Instruction Manual Tonometer-Pachymeter.

[23] Kasprzak H., Boszczyk A. (2016). Numerical Analysis of Corneal Curvature Dynamics Based on Corvis Tonometer Images. J. Biophotonics.

[24] Cicchetti D.V. (1994). Guidelines, Criteria, and Rules of Thumb for Evaluating Normed and Standardized Assessment Instruments in Psychology. Psychol. Assess..

[25] Bland J.M., Altman D.G. (1996). Statistics Notes: Measurement Error. BMJ.

[26] Bland J.M., Altman D.G. (1995). Calculating Correlation Coefficients with Repeated Observations: Part 2—Correlation between Subjects. BMJ.

[27] Luce D.A. (2005). Determining in Vivo Biomechanical Properties of the Cornea with an Ocular Response Analyzer. J. Cataract Refract. Surg..

[28] Touboul D., Roberts C., Kérautret J., Garra C., Maurice-Tison S., Saubusse E., Colin J. (2008). Correlations between Corneal Hysteresis, Intraocular Pressure, and Corneal Central Pachymetry. J. Cataract Refract. Surg..

[29] Lopes B.T., Roberts C.J., Elsheikh A., Vinciguerra R., Vinciguerra P., Reisdorf S., Berger S., Koprowski R., Ambrósio R. (2017). Repeatability and Reproducibility of Intraocular Pressure and Dynamic Corneal Response Parameters Assessed by the Corvis ST. J. Ophthalmol..

[30] Nemeth G., Hassan Z., Csutak A., Szalai E., Berta A., Modis L. (2013). Repeatability of Ocular Biomechanical Data Measurements with a Scheimpflug-Based Noncontact Device on Normal Corneas. J. Refract. Surg..

[31] Jiménez-Villar A., Mączyńska E., Cichański A., Wojtkowski M., Kałużny B.J., Grulkowski I. (2019). High-Speed OCT-Based Ocular Biometer Combined with an Air-Puff System for Determination of Induced Retraction-Free Eye Dynamics. Biomed. Opt. Express.

